# The relationship between medication possession index and functional prognosis in patients with rheumatoid arthritis: a national longitudinal cohort study from CHARLS

**DOI:** 10.1097/JS9.0000000000005048

**Published:** 2026-03-16

**Authors:** Zhongbin Xia, Wen Ye, Qianmingyue Zhang, Xinrong Pan

**Affiliations:** Health Management Center, The Second Affiliated Hospital, Jiangxi Medical College, Nanchang University, Nanchang, China

**Keywords:** functional dependence, longitudinal cohort study, medication coverage, medication possession index (MPI), rheumatoid arthritis

## Abstract

**Objectives:**

To prospectively investigate the association between the Medication Possession Index (MPI) and the risk of incident functional dependence in middle-aged and older rheumatoid arthritis (RA) patients in China.

**Methods:**

This national longitudinal cohort study utilized data from the China Health and Retirement Longitudinal Study (CHARLS). A total of 2495 RA patients (mean age 67 · 5 ± 10 · 9 years; 36 · 5% male) without baseline functional dependence were included. MPI (range: 0–1), a composite, cross-sectional measure of pharmacotherapeutic coverage for 12 chronic conditions, was calculated at baseline. Complex survey weights were incorporated in all Cox models using survey::svycoxph(), and missing covariates were addressed via multiple imputation (*m* = 10). Participants were categorized by MPI quartiles (Qa:lowest to Qd:highest). The primary outcome was incident functional dependence (assistance needed in activities of daily living/instrumental activities of daily living). Multivariate Cox proportional hazards models and restricted cubic splines (RCS) were used to assess associations and dose–response relationships.

**Results:**

Over 8530 person-years (median follow-up 3–4 years), 918 functional dependence events occurred (incidence rate: 10.8/100 person-years). Higher MPI quartiles were associated with higher functional independence survival (log-rank *P* = 1.3 × 10^–4^). Compared to Qa, Qd was associated with a 29% reduced risk in the fully adjusted model (HR = 0.71, 95% CI:0.59–0.87; *P*<0.001), with a significant inverse linear trend (*P*-trend<0.001). RCS confirmed a monotonic dose–response relationship (*P*-nonlinearity = 0.18). The association was robust across multiple sensitivity analyses.

**Conclusion:**

Higher MPI, reflecting better overall treatment coverage across multiple conditions, is independently associated with a reduced risk of functional dependence in middle-aged and older RA patients, showing a monotonic dose–response relationship. MPI shows potential as a pragmatic indicator of overall healthcare engagement for identifying high-risk patients who may benefit from interventions aimed at improving treatment coverage and health management.

## Introduction

Rheumatoid arthritis (RA) is a chronic autoimmune disease characterized by persistent joint inflammation, progressive bone destruction, and functional loss^[^1,2^]^, imposing a significant public health burden globally^[^3^]^. Adherence research in RA has largely focused on disease-modifying antirheumatic drug (DMARD)-specific metrics. By contrast, older adults frequently live with multimorbidity, and adherence behaviors may generalize across conditions. We therefore evaluated a generic, multi-condition MPI and its relationship with functional prognosis. Such a composite measure may capture broader health behavior (e.g., health literacy, access, routine) beyond RA-specific therapy, potentially improving risk stratification in multimorbid populations. In China, with population aging, RA prevalence is rising, threatening the independent living ability of middle-aged and older adults and increasing the risk of functional dependence^[^4^]^. Functional dependence – defined as needing assistance in basic [activities of daiy living (ADLs)] or instrumental activities of daily living (IADLs) – is a key indicator of long-term prognosis, quality of life, and caregiving burden in RA^[^5^]^. The core goals of RA treatment include not only controlling inflammation and relieving symptoms but also preventing disability and delaying functional dependence^[^6,7^]^. Thus, exploring factors influencing functional dependence in RA patients is critical for improving their prognosis.

The China Health and Retirement Longitudinal Study (CHARLS) is a nationally representative cohort of adults aged ≥45 years, with comprehensive data on health, function, medical behavior, and socioeconomic factors^[^8,9^]^. This study uses CHARLS data to prospectively investigate the relationship between a comprehensive measure of medication treatment coverage and functional dependence risk in middle-aged and older Chinese RA patients. To assess the breadth of treatment coverage across multiple chronic conditions in RA patients, we used a composite Medication Possession Index (MPI). In this study, MPI is defined as a cross-sectional, composite metric of pharmacotherapeutic coverage, calculated as (number of chronic conditions with current medication use)/(total number of self-reported chronic conditions). This differs from traditional longitudinal adherence metrics like the Medication Possession Ratio (MPR) or Proportion of Days Covered (PDC), which focus on drug supply continuity for a single condition over time. Derived from self-reported diagnoses and drug use, MPI is easy to compute, quantitative, and reflects the proportion of a patient’s detected chronic conditions that are under current pharmacological treatment. This approach provides a more holistic evaluation of a patient’s overall pharmacotherapy engagement beyond the traditional focus on single-disease metrics like those for DMARDs. A low MPI may indicate untreated conditions (including RA) or treatment discontinuation, potentially increasing functional dependence risk.

This study aims to clarify the association between MPI and functional dependence risk in middle-aged and older Chinese RA patients using CHARLS longitudinal data, identifying high-risk groups with low MPI to inform targeted interventions^[^10^]^.

## Materials and methods

### Study design

This research utilizes the CHARLS database, specifically employing data from the fourth wave (2018) for analysis. To evaluate the relationship between MPI and event-based functional reliance, a nationally representative cohort of RA patients was gathered using multistage-stratified sampling. The research employed baseline MPI as the principal exposure variable and functional dependence as the outcome event. Functional dependence was defined as the onset of needing assistance with at least one ADL (dressing, bathing, eating, getting in or out of bed, toileting) or IADL (doing housework, preparing hot meals, shopping for groceries, managing money, taking medications). This was assessed based on participant responses to the corresponding questions in the CHARLS questionnaire. In addition to using Kaplan–Meier curves to illustrate the variation in survival probability, a multivariate Cox proportional hazards regression model was developed to measure the hazard ratio (HR) and its 95% confidence interval (CI).

This study has been reported in accordance with the STROCSS 2025 criteria^[^11^]^.

### Research sample

The CHARLS database’s diagnosed RA patients served as the study’s subjects. RA was defined solely by an affirmative self-reported response to the question “Has a doctor ever told you that you have rheumatoid arthritis?” obtained during standardized health interviews in the CHARLS survey. No serological or clinical examination data were available for validation. The inclusion criteria were as follows: (1) Absence of functional dependence at baseline evaluation; (2) Comprehensive data available for MPI computation; (3) At least one follow-up assessment conducted. A flow diagram illustrating participant inclusion and exclusion is provided as Supplemental Digital Content Figure S1, available at: http://links.lww.com/JS9/H60 in the Supplementary Materials. The final analytical sample consisted of 2495 participants.

HIGHLIGHTS

- Higher MPI is independently associated with reduced risk of functional dependence in RA.
- A clear monotonic dose–response relationship between MPI and functional prognosis is observed.
- MPI measures overall treatment coverage across comorbidities, beyond RA-specific adherence.
- It represents a pragmatic, clinically feasible indicator for risk stratification.
- Findings are robust across multiple sensitivity and competing-risk analyses.

The MPI was calculated as the ratio of the number of chronic conditions for which a participant reported current medication use to the total number of chronic conditions detected from the same self-reported survey module. It is a cross-sectional measure of the breadth of pharmacotherapeutic coverage across comorbidities. The 12 chronic conditions included in the MPI calculation were: hypertension, diabetes, dyslipidemia, cancer, chronic lung disease, liver disease, heart disease, kidney disease, digestive disorder, psychiatric disorder, memory-related disorder, and asthma (as listed in Table 1). For each condition, “current medication use” was defined by a positive response to the corresponding CHARLS medication question: “Are you now taking any prescribed or non-prescribed treatment for this condition?” This response encompasses a heterogeneous range of treatments from over-the-counter pain relievers to prescription disease-modifying drugs. This composite measure reflects the extent of treatment coverage across a patient’s multiple chronic conditions, rather than adherence to any single medication regimen.

**Table 1:** Baseline characteristics of study participants by MPI quartiles.

Variable	Total	Quartile a	Quartile b	Quartile c	Quartile d	*P*-value
Gender (1 = Male,0 = Female)	910 (36.5%)	234 (37.3%)	225 (35.8%)	228 (36.3%)	223 (36.4%)	0.957
Han nationality (Yes/No)	2246 (90.0%)	565 (90.1%)	562 (89.5%)	544 (86.6%)	575 (94.0%)	<0.001
Hypertension (Yes/No)	1190 (47.7%)	300 (47.8%)	318 (50.6%)	284 (45.2%)	288 (47.1%)	0.281
Dyslipidemia (Yes/No)	703 (28.2%)	179 (28.5%)	181 (28.8%)	153 (24.4%)	190 (31.0%)	0.067
Diabetes (Yes/No)	357 (14.3%)	88 (14.0%)	104 (16.6%)	73 (11.6%)	92 (15.0%)	0.086
Cancer (Any) (Yes/No)	61 (2.4%)	25 (4.0%)	14 (2.2%)	4 (0.6%)	18 (2.9%)	0.001
Lung disease (Yes/No)	589 (23.6%)	164 (26.2%)	188 (29.9%)	118 (18.8%)	119 (19.4%)	<0.001
Liver disease (Yes/No)	215 (8.6%)	61 (9.7%)	61 (9.7%)	36 (5.7%)	57 (9.3%)	0.03
Heart disease (Yes/No)	706 (28.3%)	188 (30.0%)	183 (29.1%)	171 (27.2%)	164 (26.8%)	0.546
Kidney disease (Yes/No)	381 (15.3%)	121 (19.3%)	97 (15.4%)	81 (12.9%)	82 (13.4%)	0.007
Digestive disease (Yes/No)	1109 (44.4%)	301 (48.0%)	285 (45.4%)	276 (43.9%)	247 (40.4%)	0.054
Mental illness (Yes/No)	116 (4.6%)	33 (5.3%)	28 (4.5%)	19 (3.0%)	36 (5.9%)	0.093
Memory disease (Yes/No)	133 (5.3%)	40 (6.4%)	45 (7.2%)	25 (4.0%)	23 (3.8%)	0.013
Asthma (Yes/No)	249 (10.0%)	71 (11.3%)	87 (13.9%)	29 (4.6%)	62 (10.1%)	<0.001
Hypertension (Yes/No)	1.91 ± 2.06	1.84 ± 1.99	1.86 ± 2.09	1.65 ± 1.93	2.34 ± 2.24	0.216
Lung disease (Yes/No)	1.24 ± 1.65	1.42 ± 1.88	0.94 ± 1.17	1.33 ± 1.74	1.37 ± 1.82	0.379
Mental illness (Yes/No)	2.48 ± 2.61	2.86 ± 2.82	2.19 ± 2.62	2.91 ± 2.66	2.25 ± 2.49	0.751
Hospital admissions (past year, n)	0.44 ± 1.10	0.38 ± 0.92	0.44 ± 0.95	0.46 ± 1.03	0.48 ± 1.44	0.457
Night time sleep (h)	5.73 ± 2.20	5.76 ± 2.21	5.60 ± 2.21	5.72 ± 2.23	5.87 ± 2.12	0.183
Nap duration (min/day)	40.10 ± 49.91	35.17 ± 43.96	35.35 ± 47.39	41.54 ± 50.89	48.54 ± 55.69	<0.001
Vigorous activity (days/week)	1.89 ± 2.87	1.80 ± 2.80	2.03 ± 2.98	1.91 ± 2.85	1.82 ± 2.84	0.477
Moderate activity (days/week)	2.87 ± 3.18	2.83 ± 3.21	3.05 ± 3.22	2.98 ± 3.17	2.61 ± 3.13	0.077
Light activity (days/week)	5.27 ± 2.82	5.32 ± 2.80	5.39 ± 2.71	5.37 ± 2.76	4.99 ± 2.98	0.041
Social: Visit friends (Yes/No)	777 (31.2%)	175 (28.0%)	193 (30.7%)	191 (30.4%)	218 (35.7%)	0.029
Number of prescription medications (count)	1.91 ± 2.06	1.84 ± 1.99	1.86 ± 2.09	1.65 ± 1.93	2.34 ± 2.24	0.012
Social: Mahjong/Chess/Cards (Yes/No)	303 (12.2%)	84 (13.4%)	72 (11.5%)	62 (9.9%)	85 (13.9%)	0.109
Social: Provide unpaid help (Yes/No)	313 (12.6%)	63 (10.1%)	85 (13.5%)	90 (14.3%)	75 (12.3%)	0.115
Social: Dance/Exercise in park (Yes/No)	110 (4.4%)	24 (3.8%)	36 (5.7%)	23 (3.7%)	27 (4.4%)	0.269
Social: Association Participation (Yes/No)	38 (1.5%)	12 (1.9%)	9 (1.4%)	6 (1.0%)	11 (1.8%)	0.505
Social: Volunteer/Charity (Yes/No)	99 (4.0%)	21 (3.4%)	32 (5.1%)	17 (2.7%)	29 (4.7%)	0.098
Social: School/Training (Yes/No)	8 (0.3%)	3 (0.5%)	1 (0.2%)	1 (0.2%)	3 (0.5%)	0.558
Social: Other (Yes/No)	28 (1.1%)	6 (1.0%)	9 (1.4%)	4 (0.6%)	9 (1.5%)	0.439
Freq: Visit friends (ordinal)	3.36 ± 1.07	3.43 ± 1.03	3.36 ± 1.08	3.39 ± 1.04	3.28 ± 1.11	0.095
Freq: Mahjong/Chess/Cards (ordinal)	3.78 ± 0.66	3.75 ± 0.69	3.80 ± 0.62	3.81 ± 0.65	3.75 ± 0.69	0.314
Freq: Provide unpaid help (ordinal)	3.83 ± 0.51	3.87 ± 0.44	3.80 ± 0.56	3.81 ± 0.52	3.83 ± 0.51	0.132
Freq: Dance/Exercise in park (ordinal)	3.89 ± 0.52	3.91 ± 0.50	3.87 ± 0.56	3.90 ± 0.51	3.90 ± 0.51	0.632
Freq: Association participation (ordinal)	3.98 ± 0.20	3.97 ± 0.21	3.98 ± 0.22	3.99 ± 0.12	3.97 ± 0.23	0.415
Freq: Volunteer/Charity (ordinal)	3.94 ± 0.33	3.95 ± 0.28	3.93 ± 0.37	3.96 ± 0.23	3.92 ± 0.40	0.034
Freq: School/Training (ordinal)	2485 (99.7%)	623 (99.5%)	627 (99.8%)	627 (99.8%)	608 (99.5%)	0.558
Freq: Other social activity (ordinal)	3.98 ± 0.20	3.99 ± 0.15	3.97 ± 0.27	3.99 ± 0.11	3.97 ± 0.23	0.15
ADL: Dressing (ordinal)	379 (15.2%)	86 (13.7%)	94 (15.0%)	96 (15.3%)	103 (16.9%)	0.499
ADL: Bathing (ordinal)	435 (17.5%)	108 (17.3%)	112 (17.8%)	117 (18.7%)	98 (16.0%)	0.668
ADL: Feeding (ordinal)	130 (5.2%)	36 (5.8%)	34 (5.4%)	31 (4.9%)	29 (4.7%)	0.855
ADL: Bed transfer (ordinal)	406 (16.3%)	108 (17.3%)	98 (15.6%)	97 (15.5%)	103 (16.9%)	0.778
ADL: Toileting (ordinal)	605 (24.3%)	148 (23.6%)	161 (25.6%)	136 (21.7%)	160 (26.2%)	0.234
ADL: Urinary continence (Yes/No)	203 (8.1%)	56 (8.9%)	56 (8.9%)	36 (5.7%)	55 (9.0%)	0.091
IADL: Housework (ordinal)	688 (27.6%)	168 (26.8%)	164 (26.1%)	189 (30.1%)	167 (27.3%)	0.403
IADL: Meal preparation (ordinal)	525 (21.1%)	123 (19.6%)	129 (20.5%)	144 (23.0%)	129 (21.1%)	0.527
IADL: Grocery shopping (ordinal)	418 (16.8%)	100 (16.0%)	108 (17.2%)	108 (17.2%)	102 (16.7%)	0.927
IADL: Telephone use (ordinal)	436 (19.0%)	101 (17.7%)	109 (18.8%)	121 (20.8%)	105 (18.6%)	0.591
IADL: Medication use (ordinal)	248 (10.0%)	59 (9.4%)	62 (9.9%)	51 (8.1%)	76 (12.4%)	0.081
IADL: Money management (ordinal)	460 (18.5%)	123 (19.6%)	114 (18.2%)	123 (19.6%)	100 (16.4%)	0.397

ADL, Activities of Daily Living; AST, aspartate aminotransferase; CHARLS, China Health and Retirement Longitudinal Study; IADL, Instrumental Activities of Daily Living; MPI, Medication Possession Index; SD, standard deviation.

Note: Data are presented as mean ± standard deviation or *n* (%). *P*-values were derived from design-based Wald tests for continuous variables and Rao–Scott χ^2^ tests for categorical variables, accounting for complex survey design. All analyses incorporated CHARLS sampling weights. MPI quartiles [Qa (lowest coverage, reference group), Qb, Qc, Qd (highest coverage)] were defined by ranking the MPI, calculated as (number of chronic conditions with current medication use)/(total number of self-reported chronic conditions). The 12 chronic conditions included in MPI calculation are listed in the table.

It is important to note that the MPI used in this study differs from traditional medication adherence metrics (e.g., Proportion of Days Covered), which focus on the continuity of medication supply for a specific drug over time. Our MPI is designed to capture the cross-sectional breadth of treatment coverage across comorbidities and should be interpreted as an indicator of overall pharmacotherapeutic coverage and healthcare engagement.

Participants were categorized into a low treatment coverage group (MPI <0.25) and a high treatment coverage group (MPI ≥0.25). This dichotomous cutoff corresponded to the 25th percentile (the upper bound of the first quartile, Qa) of the observed MPI distribution, representing participants with medication coverage for fewer than approximately 3 of the 12 chronic conditions. This data-driven threshold was used for exploratory comparison and should be interpreted as such, not as a predefined clinical benchmark. Four quartile-based groupings (Qa: lowest coverage/reference group; Qb: lower-middle coverage; Qc: upper-middle coverage; Qd: highest coverage) were created for MPI to provide a more comprehensive analysis.

### Covariates

Based on research evidence from the CHARLS database, this study stratified and adjusted for multidimensional confounding variables. Demographic variables encompassed critical factors including age, gender, race, and marital status. In terms of lifestyle, it comprehensively covered current alcohol use, history of tobacco use, social isolation score, and frequency of social activities. At the clinical indicators level, it involved not only 12 comorbid conditions but also laboratory parameters [such as Aspartate Aminotransferase (AST), bilirubin total, protein total, etc.] and the number of prescribed medications. In addition, the study considered potential confounding factors such as nap duration and the frequency of light physical activity (Table 1). The study employed a stepwise adjustment technique to develop various analytical models during the analysis procedure. Model 1 was adjusted solely for age and gender as the baseline model; Model 2 included three significant comorbidities – hypertension, diabetes, and stroke – into Model 1; Model 3 additionally integrated the quantity of prescribed medications, AST, total bilirubin, total protein, and body weight to account for potential confounding pathways related to polypharmacy, systemic inflammation, liver function, and nutritional status (Table 2).

**Table 2:** Cox proportional hazard ratios (HR) of MPI quartile.

	Curve		Model 1		Model 2		Model 3	
	HR (95% CI)	*P-*value	HR (95% CI)	*P-*value	HR (95% CI)	*P-*value	HR (95% CI)	*P-*value
MPI quartile								
a (Lowest)	1 [Reference]		1 [Reference]		1 [Reference]		1 [Reference]	
B	1.07 (0.89,1.27)	0.475	1.06 (0.89,1.26)	0.543	1.06 (0.89,1.27)	0.486	1.07 (0.89,1.27)	0.469
C	0.91 (0.76,1.10))	0.343	0.91 (0.76,1.10)	0.332	0.92 (0.77,1.11)	0.408	0.93 (0.77,1.12)	0.437
d (Highest)	0.71 (0.59,0.87))	<0.001	0.71 (0.58,0.85)	<0.001	0.71 (0.59,0.86)	<0.001	0.71 (0.59,0.87)	<0.001
*P* for trend	0.002	0.003	<0.001	<0.001

Note: Model 1 adjusts for age and sex. Model 2 additionally adjusts for hypertension, diabetes, and stroke. Model 3 further adjusts for number of prescription medications, AST, total bilirubin, total protein, and body weight. P for trend was derived by fitting the ordinal quartile variable (1–4) as a continuous term. All analyses incorporated CHARLS complex survey weights using svycoxph() function.

### Patient and public involvement

Patients were not directly involved in study design, recruitment, or outcome definition. However, CHARLS data collection involved community-based surveys with participant input on health status and medication use, ensuring data relevance to real-world patient experiences. Results will be disseminated through peer-reviewed journals to inform clinical practice and patient care.

### Statistical analysis

**Figure 1.**
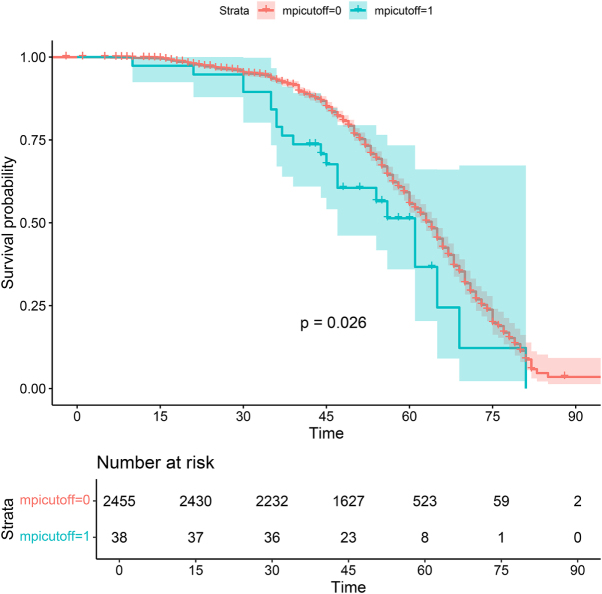
Kaplan–Meier survival curves for RA patients across MPI quartiles. Curves show functional independence survival probability over time, with significant differences between high (MPI≥0.25) and low (MPI<0.25) treatment coverage groups (log-rank *P* = 0.026). The table below lists the number of individuals at risk at different time points.

**Figure 2.**
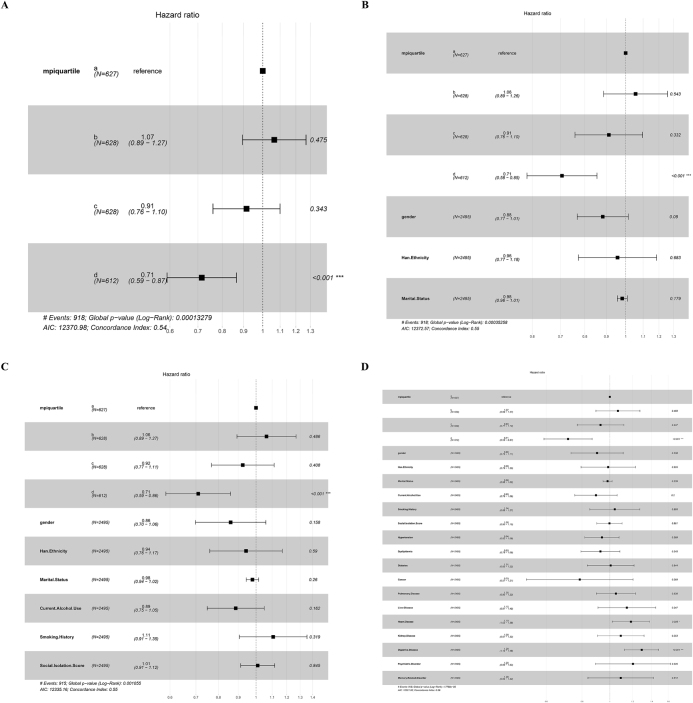
Cox proportional hazards regression forest plots for MPI quartiles and functional dependence. (A) Unadjusted model: Qd vs. Qa (HR = 0.71, 95% CI: 0.59–0.87; *P* <0.001). (B–D) Adjusted models (age/sex; + comorbidities; + laboratory parameters) show consistent 29% risk reduction in Qd. Heart disease and digestive system diseases are significant predictors in the fully adjusted model.

**Figure 3.**
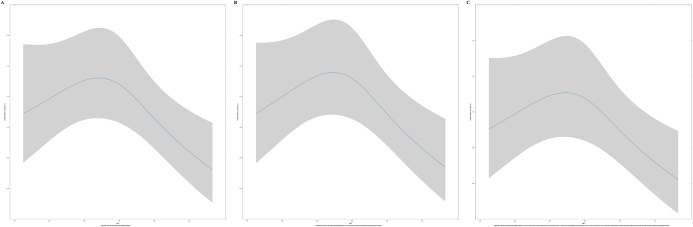
Restricted cubic spline plots of MPI and functional dependence risk. Adjusted for demographics, lifestyle, and comorbidities, plots confirm a monotonic inverse relationship (*P*-nonlinearity = 0.18) with reference at MPI = 0.25. The choice of 0.25 as the reference point was for comparability with quartile analyses and does not imply a distinct biological or clinical threshold. The shaded area represents the 95% confidence interval.

All analyses incorporated CHARLS sampling weights and clustering using the svycoxph() function from the survey package in R (version 4.3) for the Cox proportional hazards models to ensure national representativeness. This function uses Taylor linearization for variance estimation. Kaplan–Meier curves illustrate survival probabilities, and log-rank tests were employed to evaluate differences between groups (Fig. 1). Multivariate Cox proportional hazards regression was employed to estimate the (HR) and its 95% CI for MPI quartiles using a stepwise adjustment strategy (Fig. 2). To explore the dose–response relationship between MPI and functional dependency risk, restricted cubic spline (RCS) analysis was performed (Fig. 3). The MPI range for RCS was 0.2–1.0, chosen to enhance model stability by excluding extreme lower-end outliers; the overall MPI distribution ranged from 0 to 1, with the reference point set at MPI = 0.25 (HR = 1) to align with the quartile-based and dichotomous analyses for comparability. The RCS model was fitted with four knots placed at the 5th, 35th, 65th, and 95th percentiles of the MPI distribution. Missing covariates were addressed by multiple imputation with chained equations (10 datasets). Complete-case analyses produced consistent estimates with the imputed results. Furthermore, to assess the sensitivity of the chosen MPI threshold, we performed additional analyses using alternative percentile-based cutoffs (20th and 30th percentiles). The proportional hazards assumption was not violated (scaled Schoenfeld residuals, *P* > 0.05). A post hoc power analysis showed >90% power to detect a HR of 0.75 for the highest versus lowest MPI quartile, given the observed event rate and a two-sided alpha level of 0.05.

Multiple imputation vs complete-case yielded similar estimates. Alternative percentile cutoffs (20th/30th), restriction to participants with confirmed diagnosis under active treatment, exclusion of baseline IADL difficulty, and competing-risk checks all supported robustness. A summary table (Supplemental Digital Content Table S1, available at: http://links.lww.com/JS9/H60) lists HRs, 95% CIs, and *P*-values.

## Results

### Baseline characteristics

This longitudinal cohort study included 2495 patients with RA (mean age 67.5 ± 10.9 years; 36.5% male). The MPI is a composite measure of treatment coverage for 12 chronic disease medications, ranging from 0 to 1, with a median (IQR) of 0.52 (0.34–0.77). Participants were stratified into four MPI coverage quartiles (Qa: lowest coverage; Qd: highest coverage) for preliminary analysis.

Baseline characteristics differed significantly between MPI quartiles (Table 1). In terms of demographics, the Han Chinese population accounted for 90% of the entire cohort, and the prevalence increased with increasing coverage (Qc: 86.6%, Qd: 94.0%, *P* <0.001). Regarding lifestyle and social factors, the frequency of light physical activity was slightly lower in Qa (4.99 days/week) compared to Qd (5.39 days/week; *P* = 0.041). Social participation, such as visiting friends, was more common in quartiles with higher MPI (35.7% in Qd and 28.0% in Qa; *P* = 0.029). In terms of comorbidities, patients in the lower MPI quartiles had higher prevalence rates for several conditions: cancer (Qa: 4.0%, Qc: 0.6%; *P* = 0.001), pulmonary disease (Qa: 26.2%, Qc: 18.8%; *P* < 0.001), kidney disease (Qa: 19.3%, Qc: 12.9%; *P* = 0.007), and memory-related diseases (Qa: 6.4%, Qd: 3.8%; *P* = 0.013).

### Follow-up and functional dependency events

During 8530 person-years of follow-up (median follow-up time of 3–4 years per participant), 918 functional dependency events occurred (incidence rate of 10.8 per 100 person-years). Kaplan–Meier survival curves showed a clear separation between the quartiles of coverage, with an overall log-rank *P*-value of 1.3 × 10^–4^(Fig. 1). When the MPI was dichotomized at 0.25, the probability of maintaining functional independence was significantly higher in the high coverage group (MPI ≥ 0.25) compared to the low coverage group (MPI < 0.25; log-rank *P* = 0.026).

### Association between medication treatment coverage and functional outcomes

Using a stepwise adjustment strategy (see Table 2 notes), Cox proportional hazards models consistently showed a negative correlation between MPI and incident functional dependency. In the unadjusted model, compared with participants in the lowest coverage Qa (reference), those in Qb (HR = 1.07, 95% CI: 0.89–1.27; *P* = 0.475) or Qc (HR = 0.91, 95% CI: 0.76–1.10; *P* = 0.343) showed no significant association, while participants in the highest coverage Qd had a 29% reduced risk of functional dependency (HR = 0.71, 95% CI: 0.59–0.87; *P* < 0.001). This association remained robust after stepwise adjustment for covariates. A significant linear trend was observed across MPI quartiles in all models (trend *P* < 0.001) (Fig. 2D).

### Dose-response relationship

RCS analysis (Fig. 3) confirmed a monotonically negative correlation between fully adjusted MPI and functional dependency risk, with no evidence of nonlinearity (overall spline *P* = 0.18). The 95% confidence interval for the hazard ratio remained below 1 for the majority of the MPI range (MPI > 0.2), reinforcing the statistical significance of the observed inverse relationship. The reference point (MPI = 0.25) aligns with the 25th percentile used in the binary analysis, serving as a descriptive benchmark for comparative purposes rather than a definitive clinical threshold.

### Model performance and sensitivity

The model performance was acceptable, with a consistency index (C-index) of 0.58 (95% CI: 0.56–0.60) for the fully adjusted model (Model 3) (Fig. 2D). The proportional hazards assumption was not violated (scaled Schoenfeld residuals, *P* > 0.05). Additionally, sensitivity analyses employing alternative dichotomous MPI cutoffs based on the 20th and 30th percentiles yielded HRs and CIs consistent with the primary analysis using the 25th percentile (MPI = 0.25). Furthermore, a sensitivity analysis restricted to participants who reported both a physician diagnosis and current treatment for at least one chronic disease (*n* = 1918) also yielded consistent results (Model 3 HR 0.72, 95% CI 0.59–0 · 88, *P* <0.001). To address specific concerns, we performed several additional sensitivity analyses: (1) Restricting to participants with self-reported RA and current use of antirheumatic medications yielded a consistent HR (Model 3 HR 0.70, 95% CI 0.56–0.88, *P* <0.001). (2) Adding a binary covariate for “current use of any antirheumatic drug” to the fully adjusted model did not materially change the association. (3) Excluding events occurring within the first year of follow-up to reduce reverse causation yielded similar results. (4) Accounting for the competing risk of death using the Fine–Gray subdistribution hazard model produced results consistent with the primary Cox analysis. Multiple sensitivity analyses supported robustness (Supplemental Digital Content Table S1, available at: http://links.lww.com/JS9/H60).

## Discussion

This nationwide longitudinal cohort study demonstrates that a higher level of overall medication treatment coverage, as quantified by the MPI, is independently associated with a significantly reduced risk of new-onset functional dependence in middle-aged and older patients with RA. This association exhibits a clear monotonic dose–response relationship. The MPI likely serves as a proxy for a broader pattern of health engagement, access to care, and self-management behavior, which may be particularly relevant in multi-morbid older adults.

Unlike most previous studies on RA adherence, which concentrate specifically on DMARDs, our composite MPI provides a broader perspective on a patient’s overall medication management across multiple chronic conditions^[^12–14^]^. The finding that a generic MPI, encompassing medications for non-RA conditions, was strongly associated with functional prognosis suggests that it may capture a broader pattern of health engagement. It is important to acknowledge that the MPI used in this study captures the proportion of treated conditions, not the quality, necessity, or longitudinal adherence of treatment. A high MPI may reflect better overall health management, which itself is influenced by factors such as health literacy, socioeconomic status, and access to care. Therefore, the observed association may partly reflect the benefit of being more engaged in one’s overall health care^[^15^]^. Furthermore, the structure of MPI presents a conceptual limitation: a patient with multiple conditions who appropriately manages some via non-pharmacologic means (e.g., diet-controlled diabetes) will receive a lower score than a patient with only one treated condition, despite potentially more complex and effective self-management. Thus, MPI should be interpreted as a pragmatic indicator of pharmacotherapeutic coverage breadth, not as a direct measure of adherence quality or treatment appropriateness.

The dose–response relationship suggests that even modest improvements in a patient’s overall medication coverage could yield incremental benefits^[^16–18^]^. The identified MPI threshold of 0 · 25, while data-driven, offers a clinically interpretable benchmark, approximating the scenario where a patient is receiving treatment for fewer than 3 of 12 chronic conditions. While this specific cutoff requires validation, it provides a tangible starting point for clinicians to identify patients warranting more intensive support^[^19,20^]^.

From a clinical perspective, MPI offers distinct advantages as a pragmatic screening tool. MPI could be integrated into electronic health records to automatically flag patients falling below a specific threshold for targeted interventions^[^21^]^. In resource-limited settings, this simple metric could help prioritize polypharmacy reviews and adherence counseling.

However, this study has several limitations. First, the definition of RA relied solely on self-reported physician diagnosis without serological (e.g., RF, anti-CCP) or clinical examination (e.g., DAS28, joint count) confirmation. Diagnostic confusion between RA, osteoarthritis, and other rheumatic conditions in the community is possible, and the cohort likely includes misclassified cases. This inherent limitation of the CHARLS data cannot be resolved and may bias the results. Second, important RA-specific clinical variables such as disease activity scores, disease duration, and specific medication use (e.g., biologics vs. NSAIDs) were not available in CHARLS and could represent significant unmeasured confounders. The “current treatment” variable encompasses a wide spectrum from over-the-counter analgesics to disease-modifying drugs, and MPI assigns equal weight to all, which may obscure the specific impact of guideline-concordant RA therapy. Third, unmeasured confounders such as detailed socioeconomic factors and healthcare accessibility may influence the observed associations. Fourth, the MPI measures the proportion of conditions treated at a single point in time, not adherence over time. It does not distinguish between adherence to RA-specific drugs and medications for other conditions. Therefore, it should be interpreted as a marker of overall baseline treatment coverage. Fifth, MPI was measured only at baseline. Medication adherence and treatment patterns are dynamic; a single measurement may not fully capture long-term exposure and could be susceptible to reverse causality. Sixth, while we hypothesize that the association may operate through pathways such as improved control of comorbid conditions (e.g., hypertension, diabetes), reduced overall symptom burden, and sustained health-system engagement, we lacked data (e.g., consistent inflammatory markers like CRP, detailed mental health scores) to formally test these mediating mechanisms. Due to the observational nature of this study and these potential sources of confounding, causality cannot be confirmed. The findings should be interpreted as demonstrating a robust association.

## Conclusion

In summary, this nationwide cohort study found that MPI, as a composite measure of multi-morbidity treatment coverage, is independently associated with a reduced risk of functional dependence in middle-aged and older Chinese patients with rheumatoid arthritis. The close association between higher MPI and better functional outcomes, combined with its clinical feasibility, suggests that this index is a meaningful addition to risk stratification. Rather than a direct measure of medication adherence for any single condition, MPI appears to function as a pragmatic indicator of a patient’s overall engagement with health management and pharmacotherapeutic coverage. By identifying and prioritizing support for patients with low MPI, clinicians can take proactive measures that may help mitigate functional decline and maintain independence in this vulnerable population.

We recommend that future research focus on developing and evaluating targeted interventions to improve overall treatment coverage in randomized trials, specifically stratifying by low MPI. Furthermore, longitudinal studies combining objective adherence measurements, RA-specific biomarkers, and clinical activity indices are needed to validate MPI’s predictive value and elucidate the underlying biological and behavioral mechanisms linking treatment coverage to functional outcomes.

## Ethical approval

The China Health and Retirement Longitudinal Study (CHARLS) was approved by the Biomedical Ethics Committee of Peking University (approval number: IRB00001052–11015). All procedures performed in studies involving human participants were in accordance with the ethical standards of the institutional and/or national research committee and with the 1964 Helsinki declaration and its later amendments or comparable ethical standards. As this study is a secondary analysis of publicly available, de-identified data, no additional ethical approval was required.

## Consent

Informed consent was obtained from all individual participants included in the CHARLS study. Written informed consent was provided by each participant at the time of data collection.

## Guarantor

Zhongbin Xia accepts full responsibility for the work and/or the conduct of the study, had access to the data, and controlled the decision to publish.

## Data Availability

Data are from the China Health and Retirement Longitudinal Study (CHARLS), which is publicly available. Original data are available from the corresponding author upon reasonable request.
